# Perovskite With Tunable Active-Sites Oxidation State by High-Valence W for Enhanced Oxygen Evolution Reaction

**DOI:** 10.3389/fchem.2021.809111

**Published:** 2022-01-10

**Authors:** Jiabiao Yan, Mingkun Xia, Chenguang Zhu, Dawei Chen, Fanglin Du

**Affiliations:** College of Material Science and Engineering, Qingdao University of Science and Technology, Qingdao, China

**Keywords:** perovskite oxides, atom substitution, non-precious metal catalyst, oxygen evolution reaction, electrostatic spinning

## Abstract

Perovskite oxides have been established as a promising kind of catalyst for alkaline oxygen evolution reactions (OER), because of their regulated non-precious metal components. However, the surface lattice is amorphous during the reaction, which gradually decreases the intrinsic activity and stability of catalysts. Herein, the precisely control tungsten atoms substituted perovskite oxides (Pr_0.5_Ba_0.5_Co_1-x_W_x_O_3_-_δ_) nanowires were developed by electrostatic spinning. The activity and Tafel slope were both dependent on the W content in a volcano-like fashion, and the optimized Pr_0.5_Ba_0.5_Co_0.8_W_0.2_O_3_-_δ_ exhibits both excellent activity and superior stability compared with other reported perovskite oxides. Due to the outermost vacant orbitals of W^6+^, the electronic structure of cobalt sites could be efficiently optimized. Meanwhile, the stronger W-O bond could also significantly improve the stability of latticed oxide atoms to impede the generation of surface amorphous layers, which shows good application value in alkaline water splitting.

## Introduction

Electrochemical catalysis is considered as an efficient and promising strategy to convert and store sustainable energy (Chu and Majumdar, 2012). The oxygen evolution reaction (OER) is the core process and the efficiency-determining step of many electrochemical systems, such as water splitting, CO_2_ electroreduction, and rechargeable metal-air batteries (Rao et al., 2020; Li et al., 2021). The kinetic sluggishness of the OER process is the major bottleneck for improving electrolytic efficiency (Wang et al., 2021). The design of superior electrocatalysts has attracted significant attention (Zhang et al., 2020). Moreover, actual large-scale applications create the demand that a lot of work should be completed to find out economically viable non-precious metal catalysts with excellent OER performance (Li. et al., 2017; Garcia et al., 2019).

Perovskite oxides (ABO_3_) are widely used as electrocatalysts for OER considering the abundant elemental compositions, tunable structure, and low cost (Grimaud et al., 2013) (Arandiyan et al., 2021). However, their low conductivity and less active sites restrict further development (Li et al., 2020b). Strategies to solve these problems include increasing intrinsic activity, exposing more active sites, enhancing electrical conductivity, and so on (Liu. et al., 2021, Li et al., 2020). Component engineering could adjust the physical and chemical structure of perovskites to improve catalytic activity (Guo et al., 2019), which is very attractive for perovskite materials with good element compatibility (Zhang. et al., 2019). For example, Sr^2+^ doping is found to enhance the concentration of Co^4+^ on the surface of the oxide and promote the catalytic activity for OER with double perovskite oxides PrBa_1-x_Sr_x_Co_2_O_5+δ_ (Wu_ et al., 2016). The two-component (Fe and Mn) controlled La_0.6_Sr_0.4_Co_0.8_Fe_0.1_Mn_0.1_O_3-δ_ produces a higher surface oxygen vacancy (Vo) concentration and a faster oxygen ion diffusion coefficient, strengthening the participation of lattice oxygen in OER and significantly improving the activity (Tang et al., 2021). Numerous studies have shown that component optimization is feasible and promising, while most of them emphasize the improvement of activity, but ignore the regulation of stability (Chen et al., 2018).

The reasonable design of perovskite electrocatalysts should consider the optimization of both activity and stability to meet the actual application requirements (Yan et al., 2017; Song et al., 2020). Although the participation of lattice oxygen in the reaction (lattice oxygen oxidation mechanism, LOM) can break the theoretical limit of the conventional adsorbate evolution mechanism (AEM) and greatly reduce the reaction overpotential (Grimaud et al., 2017), it also leads to surface amorphization which reduces the catalytic stability, such as Ba_0.5_Sr_0.5_Co_0.8_Fe_0.2_O_3-δ_, SrCo_0.8_Fe_0.2_O_3-δ_, Pr_0.5_Ba_0.5_CoO_3-δ_, (Shao and Haile, 2004; Da et al., 2019). Therefore, research into regulating the electronic structure of active sites and preventing surface amorphization remains necessary for designing perovskite electrocatalysts (Pi et al., 2021).

Herein, we introduced high-valence W metal cations to optimize the OER performance of Pr_0.5_Ba_0.5_CoO_3_ (named PrBaCo). High-valence cations with strong electron attraction could enhance the binding ability of A or B sites to lattice oxygen to prevent surface amorphization and effectively adjust the electronic state of the active site to regulate the activity simultaneously. The catalyst with 20% W replacement exhibits superior OER activity. Moreover, reinforced covalency of the Co-O bond not only adjusts the activity but also improves the stability by inhibiting the oxidation of lattice oxygen.

## Results and Discussions

**FIGURE 1:**
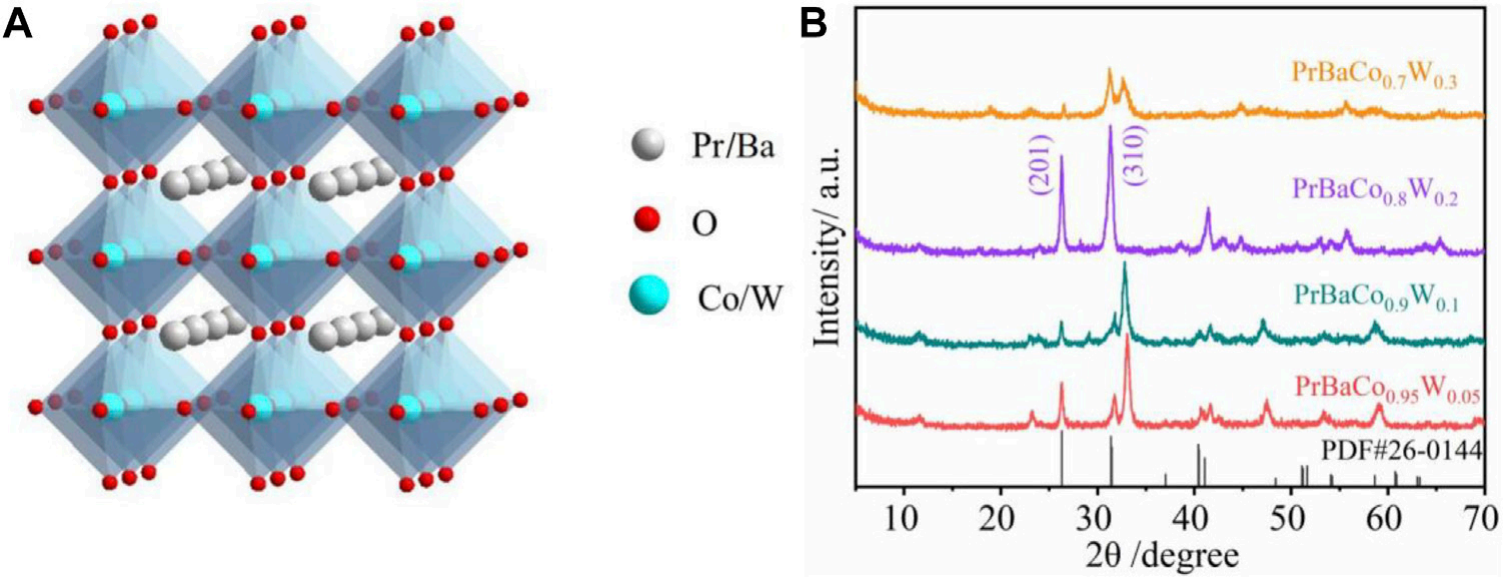
**(A)** Schematic of the perovskite crystal structure. **(B)** XRD patterns of PrBaCo_0.95_W_0.05_, PrBaCo_0.9_W_0.1_, PrBaCo_0.8_W_0.2_, and PrBaCo_0.7_W_0.3_.

**FIGURE 2:**
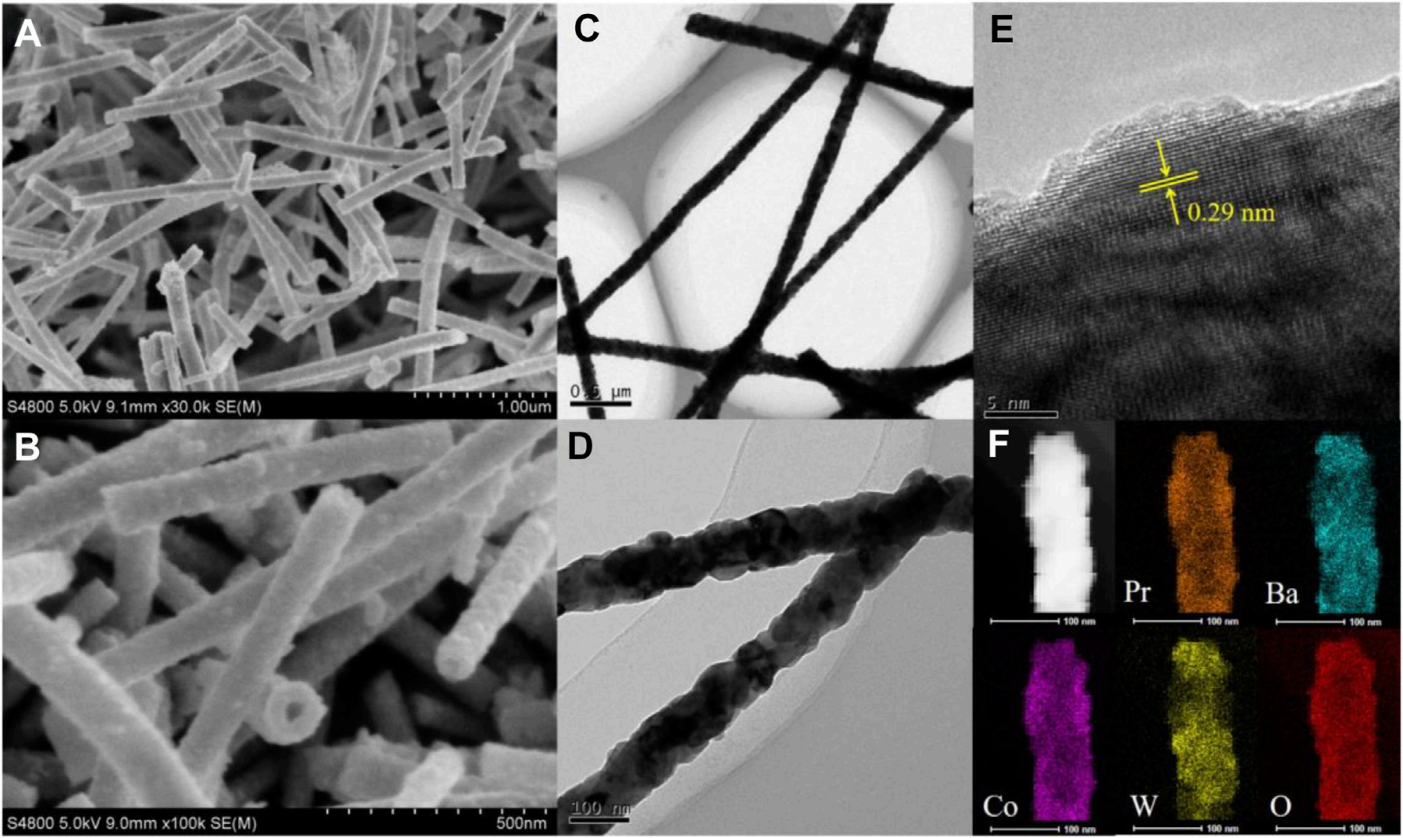
**(A, B)** SEM. **(C, D)** TEM images. **(E)** corresponding SAED patterns and **(F)** STEM-EDX mapping of PrBaCo_0.8_W_0.2_.

A series of W-substituted perovskite oxides (Figure 1A) were synthesized by precisely controlling the W content through an electrospinning method. As displayed in Figure 1B, X-ray diffraction (XRD) patterns indicated that all the catalysts (Pr_0.5_Ba_0.5_Co_1-x_W_x_O_3-δ_, *x* = 0.05, 0.1, 0.2, 0.3) good crystalline phases with the W ions successfully replaced Co ions. The diffraction peaks of Pr_0.5_Ba_0.5_Co_0.8_W_0.2_ (PrBaCo_0.8_W_0.2_) are observed at 32.8 and 26.5, corresponding to planes (310) and (201), being consistent with the PDF#26-0144. It is also then taken as an example to study the morphology and structure information. Scanning electronic microscopy (SEM) images reveal that one-dimensional nanowire is the dominant product (Figures 2A,B). Transmission electron microscopy (TEM) images are further used to give detailed morphology information. It is observed that the resulting solid product is a thin nanowire and the diameter of the nanowire is about 50 nm (Figures 2C,D). The corresponding high-resolution TEM (HRTEM) images show that the Pr_0.5_Ba_0.5_Co_0.8_W_0.2_ sample displays a single-crystalline nature and the lattice spacing is 0.29 nm, which are clearly consistent with the XRD characterization (Figure 2E). Elemental distributions were then detected by a STEM-EDX mapping, where Pr (orange), Ba (indigo), Co (purple), W (yellow), and O (red) are uniformly distributed throughout the whole nanowire (Figure 2F).

**FIGURE 3:**
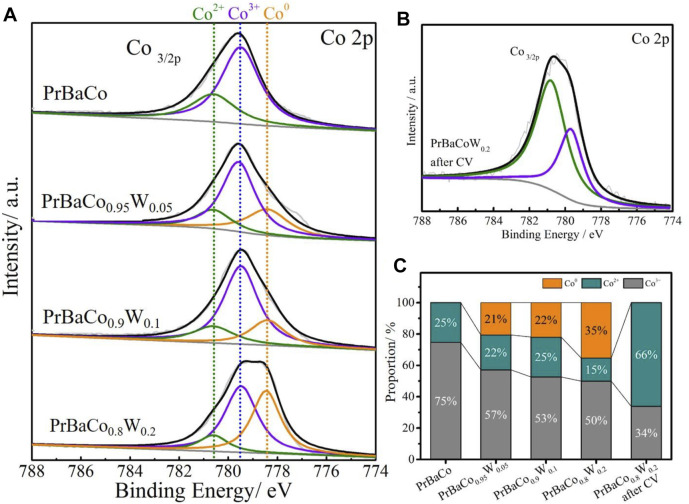
**(A)** XPS spectra (Co 2p) of PrBaCo, PrBaCo_0.95_W_0.05_, PrBaCo_0.9_W_0.1_ and PrBaCo_0.8_W_0.2_. **(B)** XPS spectra (Co 2p) of PrBaCo_0.8_W_0.2_ after CV test. **(C)** Co species proportion map of different samples.

Information on the chemical states of the electrocatalysts was examined by X-ray photoelectron spectroscopy (XPS) (Wu et al., 2019). The XPS full spectrum was provided in Supplementary Figure S1. The fine-scanned Co 2p XPS spectra of the PrBaCo, PrBaCo_0.95_W_0.05_, PrBaCo_0.9_W_0.1,_ and PrBaCo_0.8_W_0.2_ were given in Figure 3A, in which the peaks of Co 2p_3/2_ and Co 2p_1/2_ were located at around 780 and 797 eV, respectively (Tang et al., 2020). The two fitted peaks for Co 2p_3/2_ are Co^3+^ (ca. 779.5 eV) and Co^2+^ (ca. 780.8 eV) in PrBaCo. For W-substituted PrBaCo, the newly fitted peaks (778.3 eV) increased with the increasing W content (Figure 3B), corresponding to the Co^0^ (Liu et al., 2020). Taking into account the prone to surface remodeling during the OER reaction (Diaz-Morales et al., 2016), cyclic voltammetry (CV) testing was carried out to obtain a more realistic catalytic surface (Supplementary Figure S2). After CV for 100 cycles, the Co^0^ peak disappeared and the content of Co^2+^ reached up to 66% (Zhou et al., 2019), which was originated from the formation of cobalt oxyhydroxide (CoOOH) as active sites for enhanced OER (Figure 3C) (Da et al., 2019). At the lower potential, the active sites (Co) with lower valence state are more conducive to being pre-oxidated and the reconstruction of the intermediate Co-OOH structure are carried out (Xiao et al., 2020).

XPS of O 1s was also applied to further study the surface states for these perovskite catalysts (Supplementary Figure S3). In the deconvoluted O 1s spectrum (Supplementary Figure S3A), the peaks with the binding energy from 528.2 to 533.0 eV are lattice oxygen (O_L_), highly oxidative oxygen (O_O_^−^), surface adsorbed hydroxyl (O_OH_), and absorbed molecular water (O_H2O_), respectively (Zhang et al., 2019b). Supplementary Figure S3C provides the information that O_OH_ peaks become dominant with the W substitution, revealing an increase of adsorbed hydroxyl on the surface of catalysts and reinforced covalency of the Co-O bond. For PrBaCo_0.8_W_0.2_ after 100 CV cycles, highly oxidative oxygen completely disappeared and the content of adsorbed hydroxyl groups further increased (Supplementary Figure S3B). Adsorption of the active site to the hydroxyl is a key step in the OER process (Teng et al., 2020), and the hydroxyl-rich surface is undoubtedly beneficial to increase the reaction rate (Xia et al., 2020). The disappearance of highly oxidative oxygen suggests that it is difficult for lattice oxygen to participate in the OER process and become oxidized, which would prevent surface amorphization.

**FIGURE 4:**
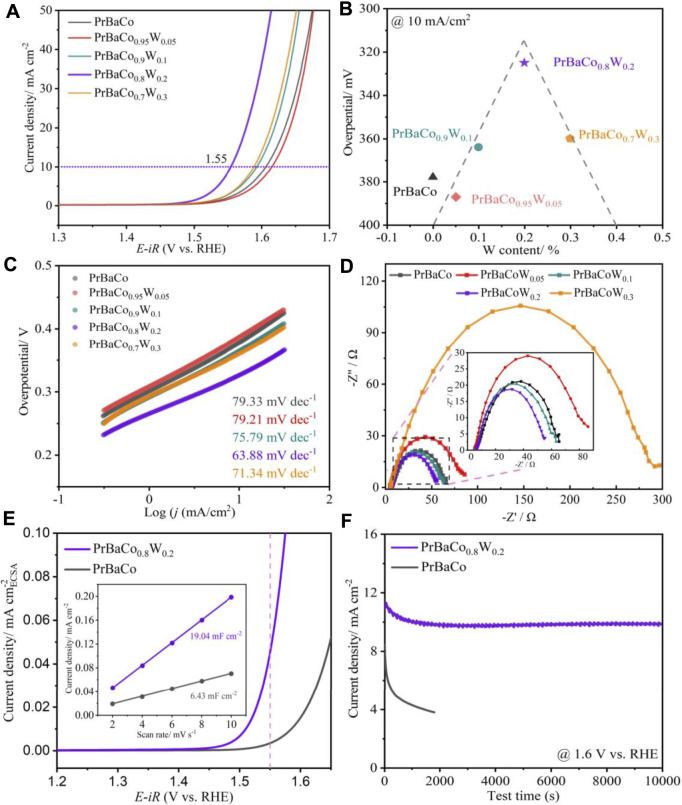
**(A)** Linear sweep voltammetry (LSV) polarization curve of various catalysts. **(B)** The volcano curves. **(C)** Corresponding Tafel slopes. **(D)** EIS plots. **(E)** LSV polarization curve normalized by the ECSA (containing double-layer capacitances of PrBaCo and PrBaCo_0.8_W_0.2_). **(F)** Chronopotentiometry curve at *j* = 10 mA/cm^2^.

The OER performance was evaluated in 1 M KOH aqueous solution at 25°C with the use of a standard three-electrode system. As shown in Figure 4A, all of the W-substituted (>10%) catalysts exhibit better OER activity in comparison to PrBaCo. When the current density reaches 10 mA/cm^2^, the potential is only 1.55 V for PrBaCo_0.8_W_0.2_, while it is 1.62 V for PrBaCo, exhibiting excellent competitiveness in the field of alkaline OER based on perovskite oxides (Supplementary Table S1). Interestingly, the electrocatalytic activity is dependent on the W content in a volcano-like fashion (Figure 4B), and PrBaCo_0.8_W_0.2_ is located near the apex of the volcano curve, demonstrating the optimal catalytic activity. To understand the mechanism for the improvement of catalytic activity, the Tafel slope was studied for the reaction kinetics (Figure 4C). Consistent with the trend of electrocatalytic activity, W-substituted perovskite oxides also show the volcano-like Tafel slope (Supplementary Figure S4). The Tafel slope of optimized PrBaCo_0.8_W_0.2_ is 63.88 mV dec^−1^, much smaller than that of PrBaCo (79.33 mV dec^−1^) and exhibiting the faster kinetics rate. Furthermore, electrochemical impedance spectroscopy (EIS) was conducted to reveal the reaction kinetics occurring at the electrolyte/electrode interface, considering the charge transfer resistance (Rct) is closely related to the OER process. As seen from Figure 4D, the Rct are 68 Ω, 85 Ω, 65 Ω, 55 Ω, 300 Ω for PrBaCo, PrBaCo_0.95_W_0.05_, PrBaCo_0.9_W_0.1_, PrBaCo_0.8_W_0.2_, and PrBaCo_0.7_W_0.3_, respectively. Among them, PrBaCo_0.8_W_0.2_ also exhibits optimal charge transfer capacity during the OER process. The information from Tafel slope and Rct suggests the improved OER reaction kinetics is due to the enhanced electron transfer through introducing appropriate W. In order to obtain the real reaction area, CV testing was used at different scan rates in the non-Faradaic potential region to acquire the double-layer capacitances (C_dl_) for PrBaCo and PrBaCo_0.8_W_0.2_ (Supplementary Figure S5 and Figure 4E). The C_dl_ of PrBaCo_0.8_W_0.2_ (19.04 mF cm^2^) is about three times larger than that of PrBaCo (6.43 mF cm^2^). Larger C_dl_ represents a larger electrochemically active area (ECSA) that exposes more active sites, which contributes to the enhancement of electrocatalytic activity. Moreover, LSV polarization curve was normalized by the ECSA to further evaluate the improvement of the intrinsic activity of PrBaCo_0.8_W_0.2_ in Figure 4E.

**FIGURE 5:**
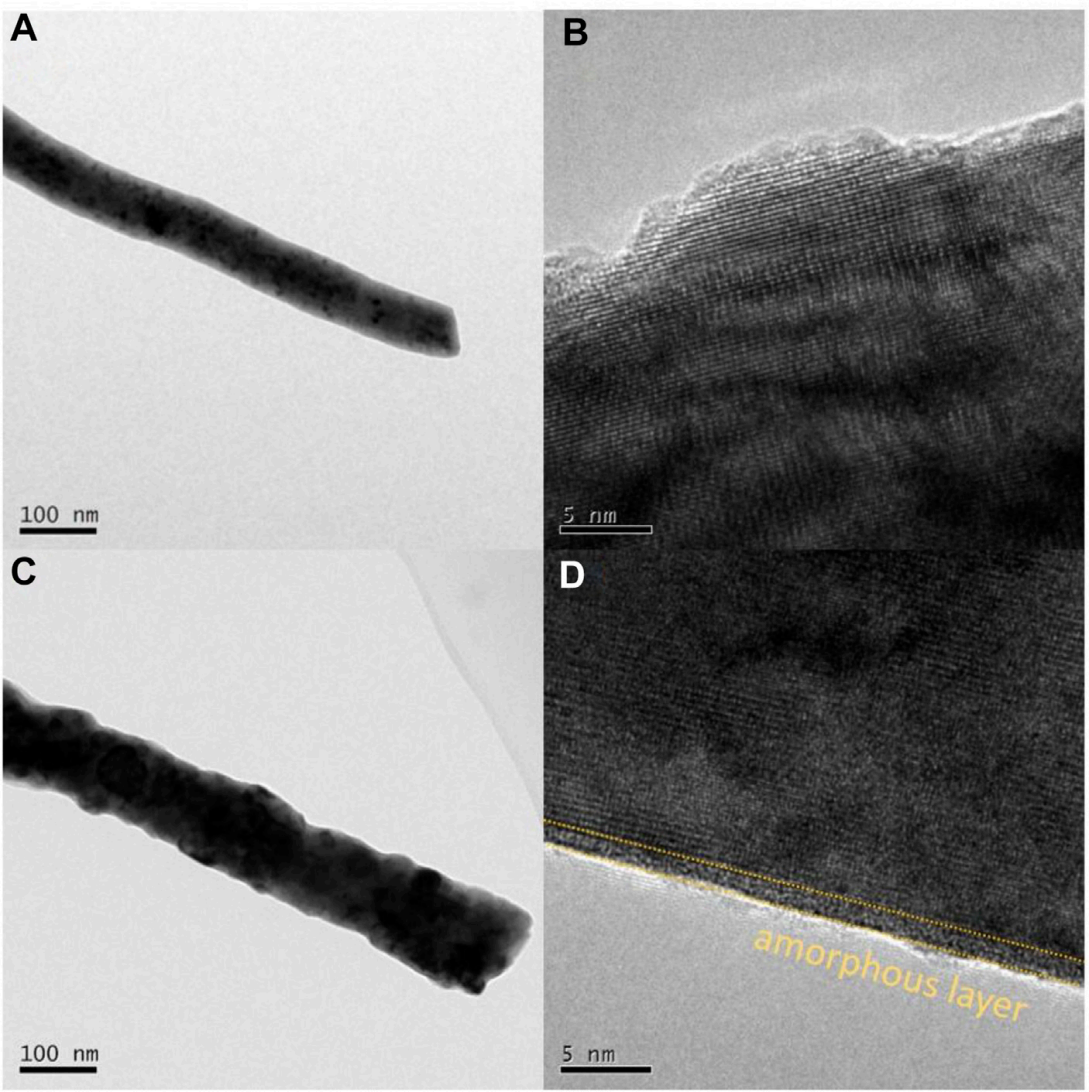
TEM images of **(A, B)** PrBaCo_0.8_W_0.2_, **(C, D)** PrBaCo_0.8_W_0.2_ after stability test.

The stability of perovskite oxide is one of the most important properties for alkaline OER, and chronopotentiometry was undertaken to assess it. As displayed in Figure 4F, the optimized PrBaCo_0.8_W_0.2_ exhibited a long-term catalytic stability (>10000s) compared with the initial PrBaCo. Additionally, the structural change of PrBaCo_0.8_W_0.2_ after the stability test was investigated by TEM images in Figure 5. It is worth noting that the one-dimensional nanostructure was well retained and there were only two to three amorphous layers on the surface of the catalyst, indicating the suppressed surface amorphous for good structural stability. All test results demonstrate that the OER performance of perovskite oxides could be productively adjusted through component engineering of W-substitution. The substitution of high-valence W cations could effectively increase the covalent between active sites and oxygen, prevent surface amorphization, and boost stability and activity.

## Conclusion

In summary, tungsten atoms substituted into perovskite oxide (Pr0.5Ba0.5Co1-xWxO3-δ) nanowires were developed by electrostatic spinning. The activity and Tafel slope were both dependent on the W content in a volcano-like fashion, and the optimized Pr_0.5_Ba_0.5_Co_0.8_W_0.2_O_3-δ_ exhibits both excellent activity (overpotential 325 mV at 10 mA cm^2^) and superior stability (10 mA cm^2^ for >10,000 s) compared with other reported perovskite oxides. The W atoms in the B site of the perovskite oxide could tune the local coordination environment for the lower valence state of Co into the active site (CoOOH) availably for enhancing the intrinsic activity. Meanwhile, it could significantly improve the lattice stability by reinforcing the covalency of the Co-O bond to impede the surface amorphous phenomenon, showing good application value in alkaline water splitting.

## Materials and Methods

### Materials

Praseodymium(Ⅲ) nitrate hexahydrate [Pr(NO_3_)_3_.6H_2_O], Barium acetate (C_4_H_6_BaO_4_), Cobalt(Ⅱ) nitrate hexahydrate [Co(NO_3_)_2_.6H_2_O] were purchased from Sinopharm Chemical Reagent Co., Ltd. Ammonium Metatungstate [(NH_4_)_6_H_2_W_12_O_40_·XH_2_O] was purchased from Aladdin Chemistry Co., Ltd. Ultra-pure water (18.25 MΩ cm) was is used to prepare all aqueous solutions in this work.

### Preparation of Catalysts

To synthesis the perovskite catalysts, Pr(NO_3_)_3_.6H2O (0.1 mmol), C4H6BaO4 (0.1 mmol), Co(NO_3_)_2_.6H_2_O (0.2-x mmol), and (NH_4_)_6_H_2_W_12_O_40_·XH_2_O (x/12 mmol), were dissolved in a solution containing DMF (5 ml) and PVP (0.8 g). Next, the solution was stirred continuously for 12 h at a speed of 500 r/min at room temperature. The obtained pick solution was transferred to an injection syringe, and then electrospun using a DC voltage of 18 kV. Finally, the resulting solid product was collected and calcined at 700°C for 6 h in air. The heating rate was 1°C/min.

### Characterizations

The structural information and properties of the catalysts were obtained by scanning electron microscopy with energy dispersive X-ray (SEM-EDX; Hitachi S-4800), transmission electron microscopy (TEM; JEOL JEM-2100F, aberration-corrected STEM: Hitachi 2700C), powder X-ray diffraction (XRD; Bruker D8 Advance), and X-ray photoelectron spectroscopy (XPS; Thermo ESCALAB 250XI).

### Electrochemical Measurements

Electrochemical measurements for OER were performed in 1 M KOH aqueous solution at 25°C on a standard three-electrode system on CHI760E electrochemical workstation (CH Instrument, United States). A glassy carbon electrode (5 mm in diameter) was used as the working electrode, and a large surface area Platinum mesh (1 × 1 cm) and a saturated calomel electrode (SCE) were used as the counter and reference electrode, respectively. All potentials measured were calibrated vs. RHE using the following equation:

E(RHE)=E+0.2224V+0.0592×pH

The catalyst (4 mg) was dispersed in ethanol (2 ml) and ultrasonicated for 15 min, followed by adding 100 µl Nafion solution (5 wt%, Sigma Aldrich, United States). The working electrode was coated with 10 µL of the good dispersity liquid and dried naturally. All potentials were converted to the reversible hydrogen electrode (RHE) to unequivocally compensate for the pH changes.

## Data Availability

The original contributions presented in the study are included in the article/Supplementary Material, further inquiries can be directed to the corresponding author.

## References

[B1] Arandiyan H. S. Mofarah S. Sorrell C. C. Doustkhah E. Sajjadi B. Hao D. (2021). Defect Engineering of Oxide Perovskites for Catalysis and Energy Storage: Synthesis of Chemistry and Materials Science. Chem. Soc. Rev. 50, 10116–10211. 10.1039/d0cs00639d 34542117

[B2] Chen D. Qiao M. Lu Y. R. Hao L. Liu D. Dong C. L. (2018). Preferential Cation Vacancies in Perovskite Hydroxide for the Oxygen Evolution Reaction. Angew. Chem. Int. Ed. 57, 8691–8696. 10.1002/anie.201805520 29771458

[B3] Chu S. Majumdar A. (2012). Opportunities and Challenges for a Sustainable Energy Future. Nature 488, 294–303. 10.1038/nature11475 22895334

[B4] Da Y. Zeng L. Wang C. Gong C. Cui L. (2019). A Simple Approach to Tailor OER Activity of SrxCo0.8Fe0.2O3 Perovskite Catalysts. Electrochimica Acta 300, 85–92. 10.1016/j.electacta.2019.01.052

[B5] Diaz-Morales O. Ferrus-Suspedra D. Koper M. T. M. (2016). The Importance of Nickel Oxyhydroxide Deprotonation on its Activity towards Electrochemical Water Oxidation. Chem. Sci. 7, 2639–2645. 10.1039/c5sc04486c 28660036 PMC5477031

[B6] Garcia A. C. Touzalin T. Nieuwland C. Perini N. Koper M. T. M. (2019). Enhancement of Oxygen Evolution Activity of Nickel Oxyhydroxide by Electrolyte Alkali Cations. Angew. Chem. Int. Ed. 58, 12999–13003. 10.1002/anie.201905501 31250499

[B7] Grimaud A. Diaz-Morales O. Han B. Hong W. T. Lee Y.-L. Giordano L. (2017). Activating Lattice Oxygen Redox Reactions in Metal Oxides to Catalyse Oxygen Evolution. Nat. Chem 9, 457–465. 10.1038/nchem.2695 28430191

[B8] Grimaud A. May K. J. Carlton C. E. Lee Y.-L. Risch M. Hong W. T. (2013). Double Perovskites as a Family of Highly Active Catalysts for Oxygen Evolution in Alkaline Solution. Nat. Commun. 4, 2439. 10.1038/ncomms3439 24042731

[B9] Guo Q. Li X. Wei H. Liu Y. Li L. Yang X. (2019). Sr, Fe Co-doped Perovskite Oxides with High Performance for Oxygen Evolution Reaction. Front. Chem. 7, 224. 10.3389/fchem.2019.00224 31069212 PMC6491708

[B10] Li B.-Q. Xia Z.-J. Zhang B. Tang C. Wang H.-F. Zhang Q. (2017). Regulating P-Block Metals in Perovskite Nanodots for Efficient Electrocatalytic Water Oxidation. Nat. Commun. 8, 934. 10.1038/s41467-017-01053-x 29038552 PMC5643308

[B11] Li C. Wang Y. Jin C. Lu J. Sun J. Yang R. (2020). Prepation of Perovskite oxides/(CoFe)P2 Heterointerfaces to Improve Oxygen Evolution Activity of La0.8Sr1.2Co0.2Fe0.8O4+δ Layered Perovskite Oxide. Int. J. Hydrogen Energ. 45, 22959–22964. 10.1016/j.ijhydene.2020.06.044

[B12] Li M. Wang Y. Zheng Y. Fu G. Sun D. Li Y. (2020). Gadolinium‐Induced Valence Structure Engineering for Enhanced Oxygen Electrocatalysis. Adv. Energ. Mater. 10, 1903833. 10.1002/aenm.201903833

[B13] Li Z. Li H. Li M. Hu J. Liu Y. Sun D. (2021). Iminodiacetonitrile Induce-Synthesis of Two-Dimensional PdNi/Ni@carbon Nanosheets with Uniform Dispersion and strong Interface Bonding as an Effective Bifunctional Eletrocatalyst in Air-Cathode. Energ. Storage Mater. 42, 118–128. 10.1016/j.ensm.2021.07.027

[B14] Liu C. Shen X. Johnson G. Zhang Y. Zhang C. Chen J. (2020). Two-dimensional Metal Organic Framework Nanosheets as Bifunctional Catalyst for Electrochemical and Photoelectrochemical Water Oxidation. Front. Chem. 8, 604239. 10.3389/fchem.2020.604239 33330399 PMC7672199

[B15] Liu D. Zhou P. Bai H. Ai H. Du X. Chen M. (2021). Development of Perovskite Oxide‐Based Electrocatalysts for Oxygen Evolution Reaction. Small 17, 2101605. 10.1002/smll.202101605 34310054

[B16] Pi Y. Shao Q. Wang J. Huang B. Hu Z. Chen C.-T. (2021). Tunable One-Dimensional Inorganic Perovskite Nanomeshes Library for Water Splitting. Nano. Energ. 88, 106251. 10.1016/j.nanoen.2021.106251

[B17] Rao R. R. Kolb M. J. Giordano L. Pedersen A. F. Katayama Y. Hwang J. (2020). Operando Identification of Site-dependent Water Oxidation Activity on Ruthenium Dioxide Single-crystal Surfaces. Nat. Catal. 3, 516–525. 10.1038/s41929-020-0457-6

[B18] Shao Z. Haile S. M. (2004). A High-Performance Cathode for the Next Generation of Solid-Oxide Fuel Cells. Nature 431, 170–173. 10.1038/nature02863 15356627

[B19] Song J. Wei C. Huang Z.-F. Liu C. Zeng L. Wang X. (2020). A Review on Fundamentals for Designing Oxygen Evolution Electrocatalysts. Chem. Soc. Rev. 49, 2196–2214. 10.1039/c9cs00607a 32133479

[B20] Tang L. Fan T. Chen Z. Tian J. Guo H. Peng M. (2021). Binary-dopant Promoted Lattice Oxygen Participation in OER on Cobaltate Electrocatalyst. Chem. Eng. J. 417, 129324. 10.1016/j.cej.2021.129324

[B21] Tang L. Zhang W. Lin D. Ren Y. Zheng H. Luo Q. (2020). The Hexagonal Perovskite Ba0.5Sr0.5Co0.8Fe0.2O3−δ as an Efficient Electrocatalyst for the Oxygen Evolution Reaction. Inorg. Chem. Front. 7, 4488–4497. 10.1039/d0qi00754d

[B22] Teng W. Huo M. Sun Z. Yang W. Zheng X. Ding C. (2020). FeCoNi Sulfides Derived from *In Situ* Sulfurization of Precursor Oxides as Oxygen Evolution Reaction Catalyst. Front. Chem. 8, 334. 10.3389/fchem.2020.00334 32432081 PMC7215084

[B23] Wang Q. Zhang Z. Cai C. Wang M. Zhao Z. L. Li M. (2021). Single Iridium Atom Doped Ni2P Catalyst for Optimal Oxygen Evolution. J. Am. Chem. Soc. 143, 13605–13615. 10.1021/jacs.1c04682 34465098

[B24] Wu T. Sun S. Song J. Xi S. Du Y. Chen B. (2019). Iron-facilitated Dynamic Active-Site Generation on Spinel CoAl2O4 with Self-Termination of Surface Reconstruction for Water Oxidation. Nat. Catal. 2, 763–772. 10.1038/s41929-019-0325-4

[B25] Wu Z. Sun L.-P. Xia T. Huo L.-H. Zhao H. Rougier A. (2016). Effect of Sr Doping on the Electrochemical Properties of Bi-functional Oxygen Electrode PrBa1−Sr Co2O5+. J. Power Sourc. 334, 86–93. 10.1016/j.jpowsour.2016.10.013

[B26] Xia L. Song H. Li X. Zhang X. Gao B. Zheng Y. (2020). Hierarchical 0D−2D Co/Mo Selenides as Superior Bifunctional Electrocatalysts for Overall Water Splitting. Front. Chem. 8, 382. 10.3389/fchem.2020.00382 32509725 PMC7248173

[B27] Xiao Z. Huang Y.-C. Dong C.-L. Xie C. Liu Z. Du S. (2020). Operando Identification of the Dynamic Behavior of Oxygen Vacancy-Rich Co3O4 for Oxygen Evolution Reaction. J. Am. Chem. Soc. 142, 12087–12095. 10.1021/jacs.0c00257 32538073

[B28] Yan D. Li Y. Huo J. Chen R. Dai L. Wang S. (2017). Defect Chemistry of Nonprecious-Metal Electrocatalysts for Oxygen Reactions. Adv. Mater. 29, 1606459. 10.1002/adma.201606459 28508469

[B29] Zhang B. Fu G. Li Y. Liang L. Grundish N. S. Tang Y. (2020). General Strategy for Synthesis of Ordered Pt 3 M Intermetallics with Ultrasmall Particle Size. Angew. Chem. Int. Ed. 59, 7857–7863. 10.1002/anie.201916260 32022378

[B30] Zhang J. Cui Y. Jia L. He B. Zhang K. Zhao L. (2019). Engineering Anion Defect in LaFeO2.85Cl0.15 Perovskite for Boosting Oxygen Evolution Reaction. Int. J. Hydrogen Energ. 44, 24077–24085. 10.1016/j.ijhydene.2019.07.162

[B31] Zhang Y. Guo Y. Liu T. Feng F. Wang C. Hu H. (2019). The Synergistic Effect Accelerates the Oxygen Reduction/evolution Reaction in a Zn-Air Battery. Front. Chem. 7, 524. 10.3389/fchem.2019.00524 31396508 PMC6663983

[B32] Zhou X. Qi W. Yin K. Zhang N. Gong S. Li Z. (2019). Co(OH)2 Nanosheets Supported on Laser Ablated Cu Foam: an Efficient Oxygen Evolution Reaction Electrocatalyst. Front. Chem. 7, 900. 10.3389/fchem.2019.00900 31998691 PMC6966496

