# Supplementary material for: Perovskite With Tunable Active-Sites Oxidation State by High-Valence W for Enhanced Oxygen Evolution Reaction
Source: Front Chem. 2022 Jan 10;9:809111. doi: 10.3389/fchem.2021.809111 (PMC8784603; doi:10.3389/fchem.2021.809111)
Supplement: Supplementary file 1 [file DataSheet1.docx]

Supplementary Material

**Perovskite with** **Tunable Active-Sites** **Oxidation State by High-Valence W for Enhanced** **Oxygen Evolution Reaction**

Jiabiao Yan, Mingkun Xia, Chenguang Zhu, Dawei Chen*^*^*, Fanglin Du

College of Material Science and Engineering, Qingdao University of Science and Technology, Zhengzhou Road 53, Qingdao, 266042, P. R. China.

Correspondence and requests for materials should be addressed to D. Chen (daweichen@qust.edu.cn).

**
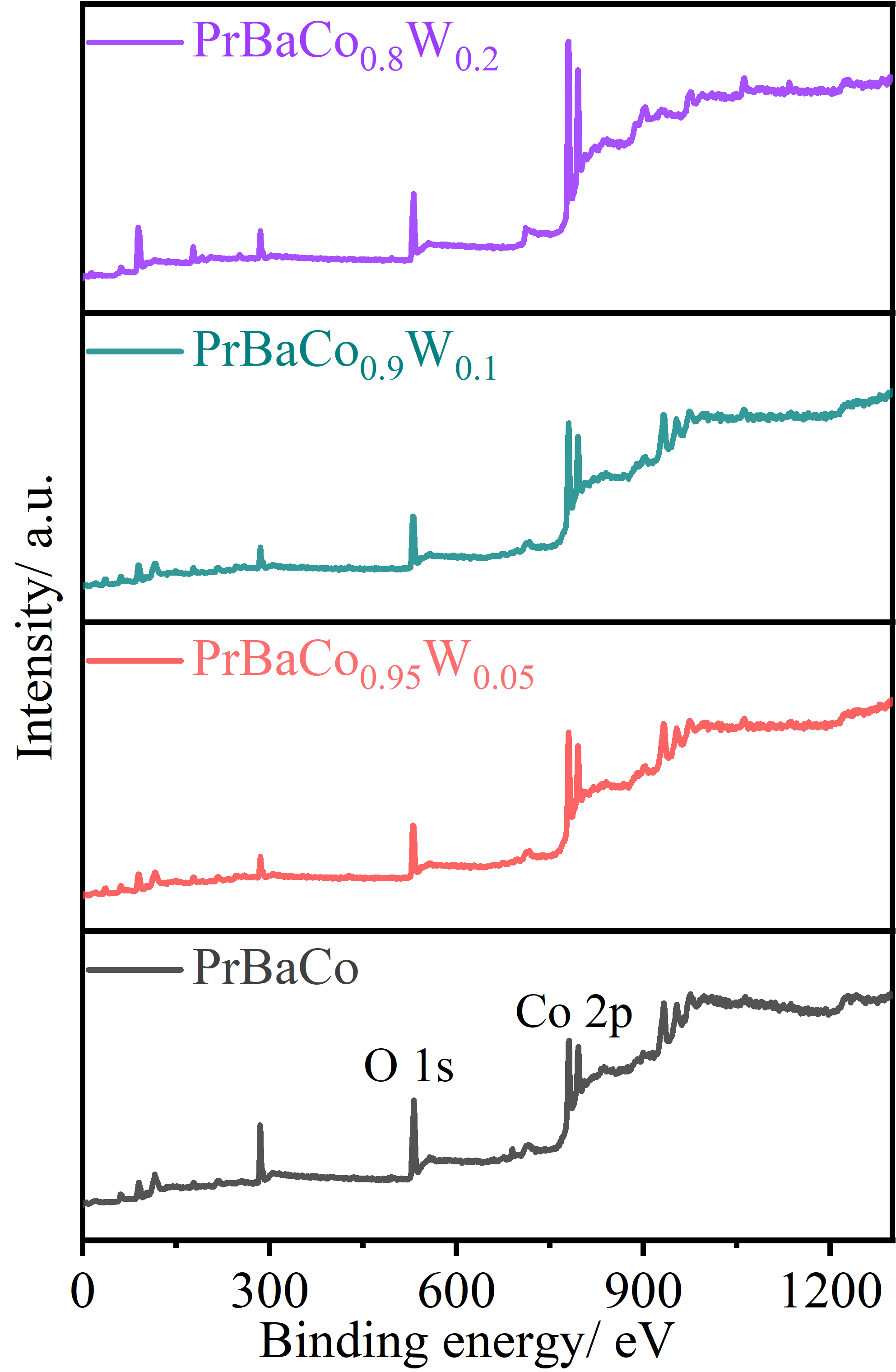
**

**Figure S1.** XPS full spectrum of PrBaCo, PrBaCo_0.95_W_0.05_, PrBaCo_0.9_W_0.1_ and PrBaCo_0.8_W_0.2_.

**Figure S2.** 100 cycles CV test of PrBaCo_0.8_W_0.2_.





**Figure S3. (A)** XPS spectra (O 1s) of PrBaCo, PrBaCo_0.95_W_0.05_, PrBaCo_0.9_W_0.1_ and PrBaCo_0.8_W_0.2_. **(B)** XPS spectra (O 1s) of PrBaCo_0.8_W_0.2_ after CV test. **(C)** O species proportion map of different samples.

**Figure S4.** The volcano curve of Tafel slopes.


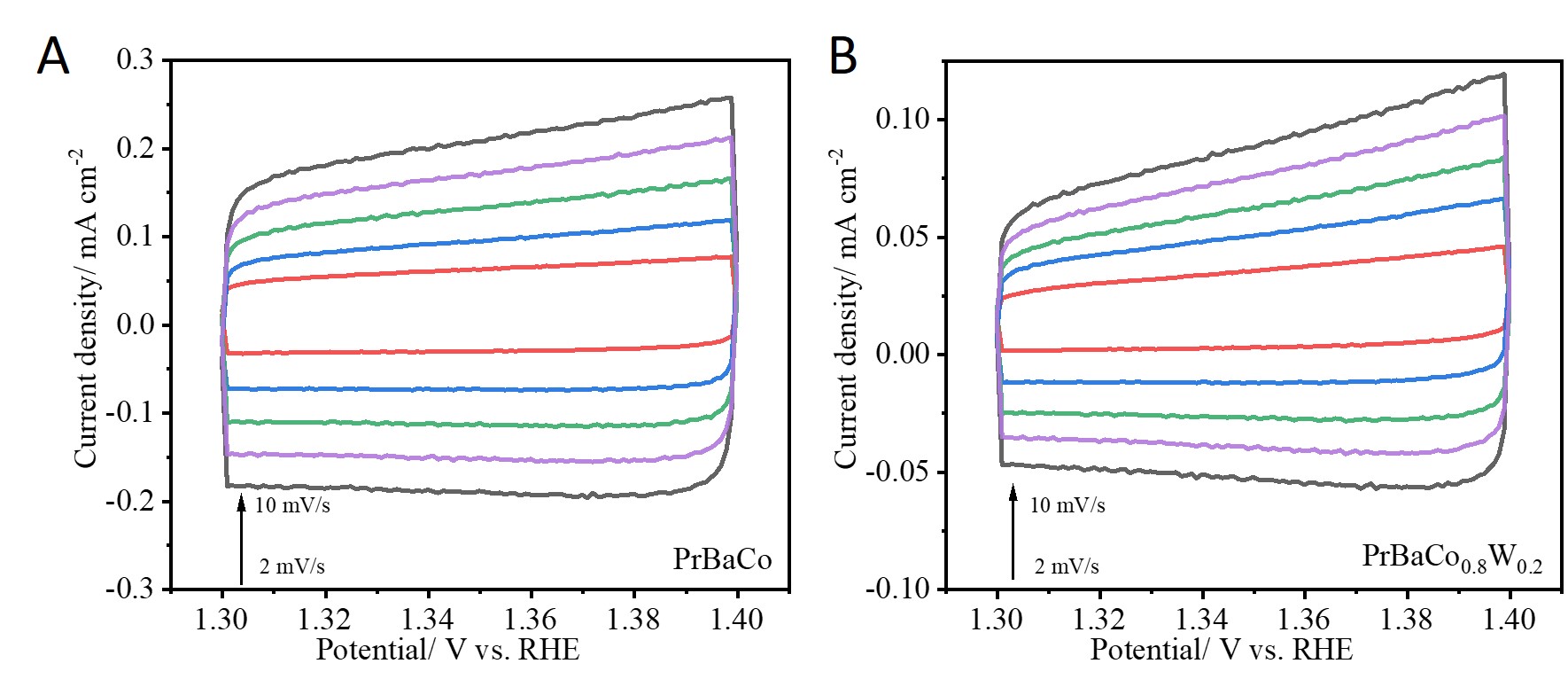


**Figure S5.** CV scanning curves in 1 M KOH electrolyte at different scan rates in non-Faradaic potential region for **(A)** PrBaCo and **(B)** PrBaCo_0.8_W_0.2_, respectively.

**Table S1.** Comparison of the OER activity for the PrBaCo_0.8_W_0.2_ with other advanced perovskite electrocatalysts reported in literature, showing the overpotential at 10 mA cm^-2^ and the electrolyte.

| Catalysts | Overpotential (mV) at 10 mA cm^-2^ | Electrolyte | Ref. |
| --- | --- | --- | --- |
| La_0.6_Sr_0.4_Co_0.8_Fe_0.1_Mn_0.1_O_3_ | 343 | 1 M KOH | (Tang et al. 2021) |
| PrBaCo_2_O_5.75_ | 360 | 1 M KOH | (Miao et al. 2019) |
| SnCo_0.9_Fe_0.1_(OH)_6_-Ar | 270 | 1 M KOH | (Chen et al. 2018) |
| Sr_0.95_Co_0.8_Fe_0.2_O_3_ | 370 | 0.1 M KOH | (Da et al. 2019) |
| LaFeO_2.85_Cl_0.15_ | 500 | 0.1 M KOH | (Zhang et al. 2019) |
| La_0.4_Sr_0.6_Ni_0.5_Fe_0.5_O_3_ | 330 | 1 M KOH | (Guo et al. 2019) |
| PrBa_0.25_Sr_0.75_Co_2_O_5.95_ | 420 | 1 M KOH | (Wu et al. 2016) |
| La_0.8_Sr_1.2_Co_0.2_Fe_0.8_O_4_ | 350 | 0.1 M KOH | (Li et al. 2020) |
| **Pr_0.5_Ba_0.5_Co_0.8_W_0.2_O_3_****_-δ_** | **325** | **1 M KOH** | **This work** |

**References**

Chen, D., Qiao, M., Lu, Y. R., Hao, L., Liu, D., Dong, C. L. et al. (2018). 'Preferential cation vacancies in perovskite hydroxide for the oxygen evolution reaction'. *Angew. Chem. Int. Ed. Engl*. 57, 8691-8696. doi: 10.1002/anie.201805520

Da, Y., Zeng, L., Wang, C., Gong, C., Cui, L. (2019). 'A simple approach to tailor OER activity of Sr_x_Co_0.8_Fe_0.2_O_3_ perovskite catalysts'. *Electrochim. Acta*. 300, 85-92. doi: 10.1016/j.electacta.2019.01.052

Guo, Q., Li, X., Wei, H., Liu, Y., Li, L., Yang, X. et al. (2019). 'Sr, Fe Co-doped perovskite oxides with high performance for oxygen evolution reaction'. *Front. Chem*. 7, 224. doi: 10.3389/fchem.2019.00224

Li, C., Wang, Y., Jin, C., Lu, J., Sun, J., Yang, R. (2020). 'Prepation of perovskite oxides/(Cofe)P_2_ heterointerfaces to improve oxygen evolution activity of La_0.8_Sr_1.2_Co_0.2_fe_0.8_O_4-δ_ Layered Perovskite Oxide'. *Int. J. Hydrogen. Energ*. 45, 22959-22964. doi: 10.1016/j.ijhydene.2020.06.044

Miao, X., Wu, L., Lin, Y., Yuan, X., Zhao, J., Yan, W. et al. (2019). 'The role of oxygen vacancies in water oxidation for perovskite cobalt oxide electrocatalysts: Are more better?'. *Chem. Commun. (Camb)*. 55, 1442-1445. doi: 10.1039/c8cc08817a

Tang, L., Fan, T., Chen, Z., Tian, J., Guo, H., Peng, M. et al. (2021). 'Binary-dopant promoted lattice oxygen participation in OER on cobaltate electrocatalyst'. *Chem. Eng J*. 417, doi: 10.1016/j.cej.2021.129324

Wu, Z., Sun, L.-P., Xia, T., Huo, L.-H., Zhao, H., Rougier, A. et al. (2016). 'Effect of Sr doping on the electrochemical properties of bi-functional oxygen electrode prba1-SrCo_2_O_5+δ_. *J. Power. Sources*. 334, 86-93. doi: 10.1016/j.jpowsour.2016.10.013

Zhang, J., Cui, Y., Jia, L., He, B., Zhang, K., Zhao, L. (2019). 'Engineering anion defect in LaFeo_2.85_Cl_0.15_ perovskite for boosting oxygen evolution reaction'. *Int. J. Hydrogen. Energ*. 44, 24077-24085. doi: 10.1016/j.ijhydene.2019.07.162
